# Identifying and predicting longitudinal trajectories of care for people newly diagnosed with HIV in South Africa

**DOI:** 10.1371/journal.pone.0238975

**Published:** 2020-09-21

**Authors:** Laura Platt, Ai Xu, Janet Giddy, Laura M. Bogart, Andrew Boulle, Robert A. Parker, Elena Losina, Ingrid V. Bassett

**Affiliations:** 1 Division of Infectious Diseases, Massachusetts General Hospital, Boston, Massachusetts, United States of America; 2 Medical Practice Evaluation Center, Massachusetts General Hospital, Boston, Massachusetts, United States of America; 3 Biostatistics Center, Massachusetts General Hospital, Boston, Massachusetts, United States of America; 4 McCord Hospital, Durban, South Africa; 5 RAND Corporation, Santa Monica, California, United States of America; 6 Center for Infectious Diseases, Epidemiology and Research, School of Public Health and Family Medicine, University of Cape Town, Cape Town, South Africa; 7 Department of Health, Provincial Government of Western Cape, Cape Town, South Africa; 8 Harvard Medical School, Boston, Massachusetts, United States of America; 9 Harvard University Center for AIDS Research, Harvard University, Boston, Massachusetts, United States of America; 10 Department of Biostatistics, Boston University School of Public Health, Boston, Massachusetts, United States of America; International AIDS Vaccine Initiative, UNITED STATES

## Abstract

**Background:**

Predicting long-term care trajectories at the time of HIV diagnosis may allow targeted interventions. Our objective was to uncover distinct CD4-based trajectories and determine baseline demographic, clinical, and contextual factors associated with trajectory membership.

**Methods:**

We used data from the Sizanani trial (NCT01188941), in which adults were enrolled prior to HIV testing in Durban, South Africa from August 2010–January 2013. We ascertained CD4 counts from the National Health Laboratory Service over 5y follow-up. We used group-based statistical modeling to identify groups with similar CD4 count trajectories and Bayesian information criteria to determine distinct CD4 trajectories. We evaluated baseline factors that predict membership in specific trajectories using multinomial logistic regression. We examined calendar year of participant enrollment, age, gender, cohabitation, TB positivity, self-identified barriers to care, and ART initiation within 3 months of diagnosis.

**Results:**

688 participants had longitudinal data available. Group-based trajectory modeling identified four distinct trajectories: one with consistently low CD4 counts (21%), one with low CD4 counts that increased over time (22%), one with moderate CD4 counts that remained stable (41%), and one with high CD4 counts that increased over time (16%). Those with higher CD4 counts at diagnosis were younger, less likely to have TB, and less likely to identify barriers to care. Those in the least favorable trajectory (consistently low CD4 count) were least likely to start ART within 3 months.

**Conclusions:**

One-fifth of people newly-diagnosed with HIV presented with low CD4 counts that failed to rise over time. Less than 40% were in a trajectory characterized by increasing CD4 counts. Patients in more favorable trajectories were younger, less likely to have TB, and less likely to report barriers to healthcare. Better understanding barriers to early care engagement and ART initiation will be necessary to improve long-term clinical outcomes.

## Background

The use of antiretroviral therapy (ART) can drastically improve outcomes for people living with HIV in low-and middle income countries [1, 2]. However, the full benefit of treatment is limited by gaps in engagement in care [3, 4]. In South Africa, estimates from 2018 reveal that while 90% of the 7.7 million patients living with HIV were aware of their diagnosis, only 62% were on treatment, with an estimated 71,000 deaths attributed to an AIDS-related illness [5]. Sustained engagement across the continuum of HIV care—from diagnosis, to linkage and retention in care, to ART-adherence and virologic suppression—will be necessary to improve clinical outcomes [6].

Current measurements of HIV care engagement tend to occur at the population level or through point-in-time assessments [7]. Such measurements obscure the impact of intermittent care interruption, transfer of care, or mortality that may be classified as lost to follow up [8].’ Group-based trajectory modeling using longitudinal data may serve as an alternative to point-in-time estimates. Using multinomial modeling, the group-based approach allows for the development of multiple distinct care trajectories from longitudinal data. Unlike growth curve modeling, which allows for individual variability around a mean population trend over time, the group-based modeling method allows for the possibility that subgroups within a population behave differently [9]. A recent study of people living with HIV in Zambia showed that care engagement can be characterized by six distinct patterns of longitudinal care, each characterized by different time frames of ART adherence/non-adherence and care engagement/interruption. Trajectories ranged from consistently high adherence and retention to early loss to follow-up without reengagement [10]. A study of patients receiving care for HIV in North Carolina defined five trajectories of care, characterized by early and late re-engagement and loss to follow-up [11]. These methods have not been applied to longitudinal HIV data from other high-prevalence settings. While these two studies were able to assess associations between trajectory membership with broad clinical and demographic factors, they did not assess other contextual factors such as social support and barriers to care.

In this study, we used group based statistical modeling to characterize CD4 trajectories for patients newly diagnosed with HIV in South Africa. CD4 count was chosen as an outcome to describe HIV care, as those engaged in care and adherent to ART (when indicated) would be expected to display a steady CD4 count increase early in treatment, followed by maintained high levels. Patients displaying different patterns of CD4 counts (failure to increase, increase followed by decrease) are likely to be experiencing lapses in care or ART adherence. We additionally sought to understand how demographic, clinical, and contextual factors correlate with longitudinal CD4 trajectories. Ultimately, these findings may be used to identify which patients may benefit from enhanced efforts for improving retention in care, and to inform what types of interventions could be most impactful.

## Methods

### Setting and study participants

Data for this study were collected through the Sizanani Trial (NCT01188941), a randomized controlled trial evaluating the use of health systems navigators to improve linkage to HIV and TB care in South Africa. This trial is described in detail elsewhere [12, 13]. Patients were approached for study enrollment by bilingual (English/Zulu) research assistants while waiting for HIV screening at two hospital-affiliated outpatient departments and two primary health clinics in the greater Durban area between August 2010 and January 2013. These clinical sites included an ART clinic affiliated with McCord Hospital in Durban, a clinic affiliated with St. Mary’s Hospital 20km west of Durban, and two additional nurse-run primary care clinics near St. Mary’s Hospital. In all, these sites spanned urban, semi-rural, and rural settings. To be eligible for study inclusion, patients had to be English or Zulu speaking adults (age ≥18) not known to be HIV infected prior to screening. We enrolled patients prior to HIV testing to minimize the impact that a new diagnosis would have on questionnaire responses. Among the 6,536 patients screened for HIV, 4,903 enrolled in the study and 1,899 (39%) were found to be living with HIV. Patient outcomes were followed longitudinally using a combination of study questionnaires, routine clinical data, and laboratory data extracted from national databases. These data sources are described in greater detail below. For inclusion in the analyses described in this paper, patients must have had two or more CD4 counts spanning greater than one year providing longitudinal data. Among patients in the Sizanani trial, 688 (36%) met this additional requirement. Children and pregnant women were excluded from the study as they have different avenues for health care delivery in South Africa.

This study was approved by McCord Hospital Medical Research Ethics Committee, St. Mary’s Hospital Research Ethics Committee, University of KwaZulu-Natal Biomedical Research Ethics committee and Partners Institutional Review Board (Protocol 2011-P-01195, Boston, MA). Participants provided written informed consent for participation. The study was monitored by an independent Data Safety Monitoring Board.

### Data collection

Upon enrollment, patients completed a baseline questionnaire that included items about demographics, emotional health, social support, and self-identified barriers to care. Demographics included patient age and gender. We measured emotional health using an adapted version of the five-item Mental Health Inventory screening test [14]. We measured social support using a 13-item questionnaire adapted from the MOS social support survey [15]. Questions regarding barriers of care were adapted from those used in the ARTAS-II trial [16]. We further categorized barriers into five domains: service delivery (wait times, treatment by staff), financial (cost of medication or transportation), perception of personal health (feeling too well or too ill to seek care), logistical (work or caretaking responsibilities), and structural (clinic hours, distance to clinic, language barriers) [17]. After survey completion, patients were tested for HIV. Those who were diagnosed with HIV had baseline CD4 counts measured and had expectorated sputum collected for mycobacterial (TB) culture. Subsequent CD4 counts were reassessed as clinically appropriate and feasible for routine HIV care.

To provide additional long-term data, we cross-matched patients with two national registries using name, gender, date of birth, and South African ID number. Cohort data was linked with data from the National Health Laboratory Services (NHLS) to obtain additional CD4 count measurements. The NHLS provides services to over 80% of the population of adults living with HIV in South Africa through a network of 265 laboratories [18]. We also cross-linked cohort data with the South African National Population Register to ascertain mortality. This register captures at least 90% of deaths nationwide [19].

### Statistical analysis

Longitudinal CD4 count was our primary parameter of interest for trajectory definition. CD4 counts were available for up to seven years after study enrollment; however, since few study participants had CD4 counts measured beyond 5 years (5%), we focused modeling on the 5 years following enrollment. Some participants had serial CD4 measurements within a short time period. To create more balanced data, we averaged serial CD4 counts such that each person had at most one CD4 measurement per year entered into the model. Given the wide range of CD4 values, we used square root transformation for model building strategies which reduced variability in the data. With mortality, patients stopped contributing CD4 measurements. We built a series of group-based trajectory models with specifications of 2 to 5 trajectory groups. We used Bayesian information criteria (BIC) to identify the model with optimal number of trajectories by calculating approximation of Bayes factor between models I and J as exp(BIC_i-BIC_j) [9, 20]. To assure that the model accurately ‘assigned’ individuals to the appropriate trajectory, we assessed the model accuracy by criteria proposed by Nagin et al.: (1) compared the estimated probability of group membership (π*j*) to the proportion classified in that group based on the highest posterior probability, (2) examined the tightness of the confidence intervals around π*j*, (3) compared the average posterior probability (AvePP) of group membership for individuals assigned to each group with 0.7 threshold, and (4) assessed whether the odds of correct classification (OCC) exceeded the minimum threshold of 5 [9]. We used Latent Class Growth Modeling (LCGM) to identify subgroups of individuals following similar patterns of CD4 count changes over time. Each of these trajectories could be specified using polynomial functions. This analysis takes into consideration heterogeneity in CD4 changes across subjects in the study.

After trajectories were unmasked, we used multinomial logistic regression to assess which baseline demographic, clinical, and contextual factors predicted membership in CD4 trajectories. To assess predictors, we used backward selection and maintained variables that showed statistically significant associations with trajectory type in the most parsimonious model. Factors considered included age, gender, the number of cohabitants in a patient’s house, a summative estimate of self-identified barriers to care, TB positivity, and ART initiation within three months of diagnosis. Barriers were counted as the number of domains (service delivery, financial, personal health perception, logistical, and structural) reported by a participant. Coefficients for risk factors indicate the increase in relative odds of being in a specific trajectory relative the reference trajectory per unit change in the risk factor.

## Results

### Cohort characteristics

Among the 1,899 HIV positive patients enrolled in the Sizanani trial, 688 (36%) had multiple CD4 measurements spanning greater than one year allowing inclusion in this analysis. The mean age of study participants was 33 years. There was an equal gender split; 337 (49%) of study participants were male. The median CD4 count at study enrollment was 218 cells/μL, with an interquartile range of 94–368. Coinfection with TB was found in 191 (28%) of participants at the time of HIV diagnosis. Among all study participants, only 108 (16%) were started on ART within three months of study enrollment. Approximately half (52%) of study participants reported no barriers to care, while a minority of participants (9%) reported barriers to care in all five domains. A total of 57 (8%) study participants died during the study period.

Compared to the patients included in this analysis, the 64% of Sizanani trial participants without multiple CD4 counts who were excluded from analysis were somewhat older (36 years-old versus 33 years-old), less likely to be married (77% versus 87%), and more likely to be employed (53% versus 45%). They had similar mean baseline CD4 counts at diagnosis (234 versus 260) and had similar rates of TB infection (27% versus 28%).

### Trajectory analysis and characterization

**Fig 1:**
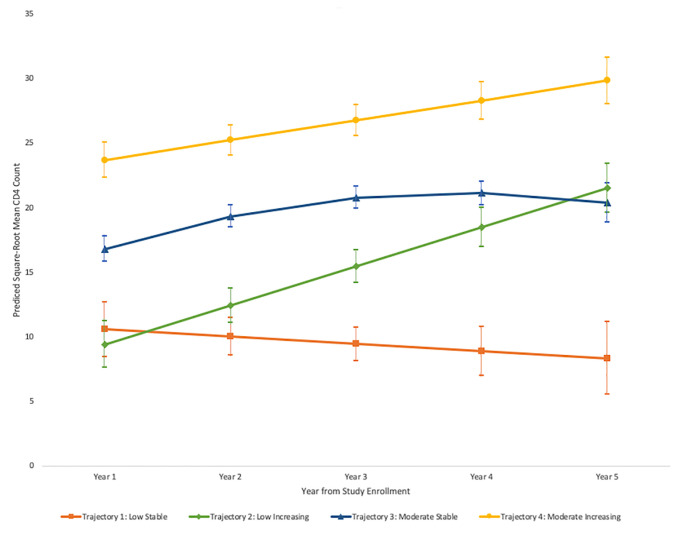
Trajectories of square root CD4 values for five years from study enrollment, as defined by group-statistical modeling. Group percentages reflect average posterior probabilities.

Using the principle of maximization of Bayesian information criteria (BIC), we determined that the model with four groups had the best fit. We named the four identified groups based upon CD4 trajectories observed: ‘1. Low Stable’, ‘2. Low Increasing’, ‘3. Moderate Stable’ and ‘4. High Increasing’ (Fig 1). Among those in the Low Stable group (23%), median CD4 counts were around 100 cells/μL at the time of study enrollment and did not rise during the five-year follow-up period. For those in the Low Increasing group (18%), median CD4 counts were around 100 cells/μL at the time of study enrollment and increased to over 300 cells/μL during the follow-up period. Those in the Moderate Stable trajectory (45%) had median CD4 counts around 300 cells/μL at enrollment that did not significantly increase during the follow-up period. Lastly, those in the High Increasing trajectory (14%) had median CD4 counts above 500 cells/μL at study enrollment with an improvement to around 900 cells/μL at the time of study completion. The average posterior probabilities for each group were greater than 0.7 and ranged from 0.73 to 0.79. Odds of correct classification, measuring improvement in membership probability of persons belonging to group 1 compared to all other groups, ranged from 4.6 for ‘Moderate Stable’ group to 19.7 for ‘High Increasing’ group. All but one odds of correct classification were greater than 5, suggesting a reasonable fit for the model.

Distribution of participants’ characteristics in each trajectory is presented in Table 1. Participants in the Low Stable trajectory were more likely to be male, more likely to live alone, and less likely to have a high school education. These participants were the most likely to pass away prior to study completion. Participants in the Low Increasing trajectory were most likely to have foregone healthcare in order to meet basic needs in the year prior to study entry. There was a higher TB prevalence among participants in the Low Stable and Low Increasing trajectories. Participants in the Moderate Stable and High Increasing trajectories were least likely to have had healthcare use in the year prior to study entry.

**Table 1:** Baseline patient characteristics stratified by estimated trajectory group^a^.

	Study total	Trajectory 1: Low Stable CD4	Trajectory 2: Low Increasing CD4	Trajectory 3: Moderate Stable CD4	Trajectory 4: High Increasing CD4
	(n = 684)^b^	(n = 158, 23%)	(n = 125, 18%)	(n = 305, 45%)	(n = 96, 14%)
Age, n (%)					
</ = 25 years	553(80.9)	138(87.3)	120 (96.0)	227 (74.4)	68 (70.8)
> 25 years	131 (19.2)	20 (12.66)	5 (4.0)	78 (25.6)	28 (29.2)
Gender, n (%)					
Female	347 (51.0)	51 (32.3)	61 (48.8)	169 (55.4)	66 (68.8)
Male	337 (49.0)	107 (67.7)	64 (51.2)	136 (44.6)	30 (31.3)
Marital Status, n (%)					
Never married	597 (87.3)	142 (89.9)	110 (88.0)	259 (84.9)	86 (89.6)
Currently married	68 (9.9)	13 (8.2)	10 (8.0)	36 (11.8)	9 (9.4)
Separated/divorced	19 (2.8)	3 (1.9)	5 (4.0)	10 (3.3)	1 (1.0)
Number of cohabitants, n (%)					
0	73 (10.7)	29 (18.4)	12 (9.6)	23 (7.5)	9 (9.4)
1	171 (25.0)	41 (26.0)	36 (28.8)	74 (24.3)	20 (20.8)
>/ = 2	439 (64.2)	88 (55.7)	76 (60.8)	208 (68.2)	67 (69.8)
Missing	1 (0.2)		1 (0.8)		
Education, n (%)					
Primary school or less	91 (13.3)	33 (20.9)	20 (16.0)	33 (10.8)	5 (5.2)
Some high school or greater	593 (86.7)	125 (79.1)	105 (84.0)	272 (89.2)	91 (94.8)
Clinic site, n (%)					
Rural/semi-rural	583 (85.2)	139 (88.0)	90 (72.0)	271 (88.9)	83 (86.5)
Urban	101 (14.8)	19 (12.0)	35 (28.0)	34 (11.5)	13 (13.5)
Healthcare use in prior year, n (%)					
0 times	77 (11.3)	11 (7.0)	6 (4.8)	46 (15.1)	14 (14.6)
1–2 times	228 (33.3)	57 (36.1)	40 (32.0)	94 (30.8)	37 (38.5)
3–5 times	249 (36.4)	54 (34.2)	55 (44.0)	109 (35.7)	31 (32.3)
> 5 times	130 (19.0)	36 (22.8)	24 (19.2)	56 (18.4)	14 (14.6)
Visit to traditional healer in prior year, n (%)					
Yes	249 (36.4)	47 (29.8)	51 (40.8)	124 (40.7)	27 (28.1)
No	435 (63.6)	111 (70.3)	74 (59.2)	181 (59.3)	69 (71.9)
Went without healthcare for basic needs, n (%)					
Yes	145 (21.1)	37 (23.4)	38 (30.4)	55 (18.0)	15 (15.6)
No	539 (78.8)	121 (76.6)	87 (69.6)	250 (82.0)	81 (84.4)
Went without basic needs for healthcare, n (%)					
Yes	102 (14.9)	27 (17.1)	25 (20.0)	38 (12.5)	12 (12.5)
No	582 (85.1)	131 (82.9)	100 (80.0)	267 (87.5)	84 (87.5)
Number of reported barriers to care, n (%)					
0	357 (52.2)	63 (39.9)	52 (41.6)	184 (60.3)	58 (60.4)
1–3	149 (21.8)	38 (24.1)	26 (20.8)	66 (21.6)	19 (19.8)
4–6	99 (14.5)	31 (19.6)	21 (16.8)	37 (12.1)	10 (10.4)
>7	79 (11.5)	26 (16.5)	26 (20.8)	18 (5.9)	9 (9.4)
Number of domains identified, n (%)					
0	357 (52.2)	63 (39.9)	52 (41.6)	184 (60.3)	58 (60.4)
1	79 (11.6)	19 (12.0)	13 (10.4)	38 (12.5)	9 (6.4)
2	46 (6.7)	12 (7.6)	8 (6.4)	19 (6.2)	7 (7.3)
3	71 (10.4)	21 (13.3)	12 (9.6)	30 (9.8)	8 (8.3)
4	72 (10.5)	28 (17.7)	20 (16.0)	18 (5.9)	6 (6.3)
5	59 (8.6)	15 (9.5)	20 (16.0)	16 (5.3)	8 (8.3)
TB at diagnosis, n (%)					
Yes	191 (27.9)	69 (43.7)	81 (64.8)	23 (7.5)	18 (18.8)
No	493 (72.1)	89 (56.3)	44 (35.2)	282 (92.5)	78 (81.3)
ART initiation within 3 months, n (%)					
Yes	108 (15.8)	4 (2.5)	63 (50.4)	34 (11.1)	7 (7.3)
No	576 (84.2)	154 (97.5)	62 (49.6)	271 (88.9)	89 (92.7)
Death prior to study completion, n (%)					
Yes	57 (8.3)	35 (22.2)	5 (4.0)	15 (4.9)	2 (2.1)
No	627 (91.7)	123 (77.8)	120 (96.0)	290 (95.1)	94 (97.9)

Baseline demographic, contextual, and clinical factors for entire cohort and stratified by estimated trajectory group.

^a^. Group assignment is made according to the maximum posterior probability assignment rule

^b^. Among the 688 participants included in the analysis, four could not be assigned a trajectory based upon CD4 counts, due to missing data in one of predictor variables.

### Predictors of trajectory membership

Table 2 depicts the impact of specific patient factors on group membership, using group 2 (Low Increasing) as the reference group. We found that, compared to our reference group, patients in groups 3 and 4 were younger (odds ratio of age < 25 years 6.88 and 7.19 for groups 3 and 4, respectively) and less likely to have TB at the time of HIV diagnosis (odds ratio 0.09 and 0.18 for groups 3 and 4, respectively). Those in groups 3 and 4 endorsed fewer barriers to care. 60% of participants in group 3 and 65% of participants in group 4 reported zero barriers to care, while 30% of participants in group 2 reported zero barriers to care. Participants in these groups were less likely to start ART within 3 months of study enrollment (odds ratio 0.12 and 0.07 for groups 3 and 4, respectively). On the other hand, there were few statistically significant differences between participants in group 1 (Low Stable) and group 2 (Low Increasing). Participants in group 1 had 20 times lower odds of starting ART within three months of study enrollment. No other factors under consideration, including demographic, clinical, and contextual variables reached statistical significance.

**Table 2:** Predictors of trajectory assignment (reference group trajectory 2, low increasing).

	Trajectory 1 (Low stable)	Trajectory 3 (Moderate stable)	Trajectory 4 (High increasing)
	Odds Ratio (95% CI), p-value	OR (95% CI), p-value	OR (95% CI), p-value
Age (<25)	3.77 (0.61–23.11), 0.15	6.88 (1.24–38.35), 0.28	7.18 (1.33–38.61), 0.02
Male Sex	1.82 (0.67–4.95), 0.24	0.88 (0.33–2.37), 0.81	0.54 (0.21–1.35), 0.19
TB coinfection	0.55 (0.19–1.61), 0.28	0.09 (0.03–0.49), < 0.01	0.18 (0.06–0.51), < 0.01
ART started within 3 months	0.05 (0.01–0.23), < 0.01	0.12 (0.03–0.49), <0.01	0.07 (0.02–0.26), < 0.01
Number of domains of barriers to care	0.82 (0.63–1.07), 0.15	0.66 (0.51–0.85), < 0.01	0.63 (0.49–0.82), < 0.01

## Discussion

This study found that group-based statistical modeling can be applied to data from longitudinal HIV care to characterize distinct CD4 trajectories. Among the patients included in this analysis, approximately 60% were in more favorable trajectories with median CD4 counts at or above 300 cells/μL throughout the study period. Patients in more favorable trajectories were younger, less likely to have TB, and reported fewer barriers to care. Among the patients who had low CD4 counts at the time of diagnosis (groups 1 and 2), approximately half failed to improve throughout the study period (group 1). Members of this group were twenty times less likely to initiate ART within three months of study enrollment compared to those whose CD4 counts improved. These results highlight the ongoing need for early diagnosis and the importance of linkage to ART following diagnosis. Further research is needed to better identify causes of late presentation and suboptimal linkage to care.

We identified four trajectories of HIV care using group-based statistical modeling. These findings are somewhat different from those of trajectory modeling in other HIV care settings. In a study using state-wide surveillance data from North Carolina to characterize care engagement, five trajectories were identified [11]. Some trajectories had patterns similar to ours (consistently high engagement and consistently low engagement). Those with changing levels of care engagement were categorized as early improvement, late improvement and steady decline. In a study of HIV care in Zambia using pharmacy and loss to follow up data, six trajectories of ART adherence and care retention were defined [10]. These trajectories were characterized by different time frames for adherence, nonadherence, loss to follow up, and reengagement. The different trajectories defined in each study may reflect differences in study population and setting, criteria for study entry, and endpoints used for trajectory definition. Trajectories for care of patients in the United States may differ from those of patients in South Africa given the dissimilar infrastructure and social context of care. The North Carolina study used lab frequency as their end point, which may be expected to decrease over time for patients with stable or improving CD4s. The study in Zambia, while in a setting more similar to ours, captured different points along the HIV care continuum by enrolling patients only after ART was initiated. In contrast, we enrolled participants prior to HIV testing and only 15% of our patients started ART within 3 months of study enrollment. The goal of unmasking trajectories is not simply to identify discrete groups of individuals, but rather to characterize the continuous distribution of changes in CD4 counts over time using a finite number of groups that approximate the range of responses. By depicting the variability in the population of people newly diagnosed with HIV in South Africa, we are better suited to pose questions and devise solutions that may help optimize patient outcomes.

Our reference group was characterized by advanced disease at study enrollment with a median CD4 count of 79 cells/μL. Compared to patients in trajectories with more preserved CD4 counts at enrollment, those in our reference group were older, more likely to have TB, and more likely to endorse barriers to health care. Characteristics of Sizanani patients with very low CD4 counts (<100 cells/μL) at HIV diagnosis have previously been described elsewhere [21]. Our demographic findings about people presenting with advanced HIV are largely similar to those in other studies in sub-Saharan Africa. In a study of over 12,000 patients newly diagnosed with HIV in South Africa, risk factors for late presentation (CD4 <200) included older age and male sex [22]. A study in Mbarara, Uganda found that older age, male gender, lower education, unemployment, and distance from clinic were all risk factors for late presentation [23]. A study in Kampala, Uganda found that older age, fewer sexual partners, and having received care from traditional healers were associated with late presentation [24].

Among the two groups presenting with low CD4 counts, one group showed improvements in CD4 counts over the study period and one did not. The only measured factor that distinguished these two groups was ART initiation within three months. This finding highlights both the importance of early ART and the ongoing issue of pre-ART loss from care. Previous studies have also highlighted both the poor outcomes associated with delayed ART initiation and the high rates of pre-ART loss from care. A study in Durban in 2006 found that 16% of patients were lost to care prior to starting ART, and among them mortality exceeded 30% within one year [25]. A study of patients following ART eligibility in Uganda found that over one third of patients had not started ART within three months and nearly one fifth did not start within one year [26]. Efforts are still needed to improve early linkage to care.

No other differences distinguished groups 1 and 2, including the number of barriers to care, despite ascertainment of many demographic, clinical, and contextual factors at study enrollment. This may partly be explained by the contradictory ways that contextual factors have been observed to impact HIV care in other studies. For example, while low education was seen as risk factor for loss to care prior to ART initiation in Uganda, having a college or university education was associated with loss to follow-up in Zambia [10, 26]. Employment has been observed as a facilitator for care engagement in some settings, and a risk factor for late presentation and delayed ART in others [21, 26, 27]. Some studies have found that care in urban setting is associated with worse HIV outcomes, while others have found distance from clinic a risk factor for poor outcomes [21–23]. Some studies have found alcohol use a risk factor for late presentation while others found it predictive of earlier presentation [23, 28]. It is thus possible that our summative measure of barriers failed to capture the complex circumstances impacting HIV care. We also note that many Sizanani study patients were excluded from this analysis due to having fewer than two CD4 counts measured during our follow-up period. It is possible that the impact of barriers would be greater for those who did not undergo repeat CD4 count testing.

Strengths of this study include ascertainment of a broad range of demographic, clinical, and contextual factors from patients at the time of HIV diagnosis, and cross-matching study patients with national datasets to improve outcome ascertainment. A limitation of this study is the exclusion of 64% of Sizanani participants from trajectory analysis due to a lack of two CD4 measurements spanning more than one year during the follow-up period. While this loss is large, it is consistent with other studies. In a study of over 12,000 patients newly diagnosed with HIV in South Africa, over a third did not have a single CD4 count measured [22]. In a systematic review of retention in care in sub-Saharan Africa, one- to two-thirds of patients are lost from care between HIV diagnosis and CD4 count measurement, and approximately one third of patients with a CD4 count were lost to care prior to starting ART [7]. We also note that outcome ascertainment in this study involved different data sources. While some outcomes such as ART initiation were measured only at study sites, CD4 count and mortality were supplemented from national datasets. We thus likely underestimated ART initiation rates.

This study found that group-based trajectory modeling adds information to longitudinal HIV data not captured with point-in-time assessments. In our study, we found that after diagnosis, most patients could be characterized by one of four trajectories. These trajectories demonstrated multiple areas for improved HIV care, including earlier diagnosis and improved linkage to care. Further work is needed to identify risk factors for early loss from care and poor ART uptake after diagnosis.
